# How a Developing Country Faces COVID-19 Rehabilitation: The Chilean Experience

**DOI:** 10.3389/fpubh.2022.924068

**Published:** 2022-07-06

**Authors:** Rodrigo Torres-Castro, Ximena Neculhueque-Zapata, Katherina Hrzic-Miranda, Ruvistay Gutiérrez-Arias, Raúl Valenzuela-Suazo, Cristobal Castro-Acuña, Marianela Ríos-Quevedo, Camilo Águila-Villanueva, Pamela Seron

**Affiliations:** ^1^Department of Physical Therapy, University of Chile, Santiago, Chile; ^2^Department of Rehabilitation and Disability, Subsecretary of Public Health, Ministry of Health, Santiago, Chile; ^3^Servicio de Medicina Física y Rehabilitación, Instituto Nacional del Tórax, Santiago, Chile; ^4^Exercise and Rehabilitation Sciences Laboratory, Faculty of Rehabilitation Sciences, School of Physical Therapy, Universidad Andres Bello, Santiago, Chile; ^5^Internal Medicine Department and CIGES, Faculty of Medicine, Universidad de La Frontera, Temuco, Chile

**Keywords:** COVID-19, rehabilitation, health policy, health services, developing country

## Abstract

The coronavirus 19 (COVID-19) pandemic has been one of the most significant challenges to public health in recent decades. The heterogeneity of government responses and the varying preparedness of health systems has determined that the pandemic's impact differs from country to country. Chile is no stranger to the challenges posed by rehabilitation in a developing country. We aimed to describe the approach to rehabilitation during the pandemic in Chile in the public health system since rehabilitation is considered a relevant health strategy from the prevention to management of complications, mitigation of sequelae, or new complications associated with COVID-19. For this, a descriptive study was conducted on the rehabilitation strategies implemented by Chile to respond to the COVID-19 pandemic. The analysis includes the context of the Chilean health system and the matrix of access to rehabilitation services in COVID-19. The Health Ministry (MINSAL) rehabilitation strategy includes five central axes: approaches, specific lines, transversal lines, intervention, and funding. Additionally, the policies were based and supported by the WHO recommendations. Intensive care unit beds were increased approximately 68%, and the primary care response was the reconversion of function depending on the epidemiological context. During the 2021–2022 period, the estimated number of people diagnosed with a post-COVID-19 condition was 80,528. With this, we can conclude that a developing country has managed to coordinate a rehabilitation policy for people with COVID-19 by generating a structure of the different health system levels. However, the effectiveness of this policy will need to be evaluated in the future.

## Introduction

The coronavirus 19 (COVID-19) pandemic, caused by the appearance of the SARS-CoV-2 virus, has been one of the most significant challenges to public health in recent decades (1). About 20% of patients develop severe disease, and ~5% require critical care (2). Those who survive critical illness have a significant probability of developing short-, medium- and long-term sequelae that can affect their functioning and quality of life (3, 4). Additionally, a group of those not hospitalized will also develop important sequelae that can affect their quality of life (5).

So far, the literature has shown a large number of physical, respiratory, and mental health sequelae, among others (3, 6). Although the disease is primarily respiratory, it significantly affects other systems, such as cardiovascular or neurological, and the populations most at risk of developing the severe disease are those with comorbidities and advanced age (7, 8). Persistent symptoms such as fatigue and dyspnea are commonly reported in post-COVID-19 patients after hospital discharge (9). Additionally, a decreased functional capacity and difficulties with the activities of daily living such as mobility and self-care were also identified in patients, particularly older adults and people with comorbidities (3).

Faced with this wave of patients with sequelae that affected their quality of life, terms such as long COVID, long-haul COVID, or chronic COVID syndrome have appeared, which define the sequelae that appeared after infection by SARS CoV-2 (4, 10–12). However, in October 2021, the WHO defined the concept of post-COVID-19 as: “A condition occurs in individuals with a history of probable or confirmed SARS-CoV-2 infection, usually 3 months from the onset of COVID-19 with symptoms and that last for at least 2 months and cannot be explained by an alternative diagnosis” (13). This is a significant concern as people hospitalized for COVID-19 are at risk of developing post-COVID-19 condition in more than 20% of cases (14).

Pulmonary rehabilitation (PR) is based on an interdisciplinary and comprehensive intervention that includes aerobic and resistance training and other adjuvant interventions such as education, management of nutritional aspects, or psychology (15). In patients recovering from COVID-19 contribute to improving physical capacity, symptoms and quality of life (16). However, their implementation is heterogeneous, particularly in Latin America (17).

In many countries, health centres have been overwhelmed by a sudden increase in cases, especially patients requiring intensive care (18). At the time, the most pragmatic recommendation was to advise physical distancing to minimize person-to-person transmission and thereby flatten the epidemic curve (18, 19). The main objective of these measures was to prevent a more rapid spread of COVID-19 and allow more time for public health and medical care services to be better prepared for the prevention and management of the disease (18). On the other hand, healthcare workers (HCW) who work in rehabilitation also had to face consequences, particularly in mental health. HCWs, particularly women, have a high risk for depression, anxiety, and stress during the COVID-19 pandemic (20).

Additionally, at the beginning of the pandemic, it was impossible to carry out rehabilitation programs in many countries as health systems were under stress, and the infrastructure of physical medicine and rehabilitation services was used to care for acute patients (21). In addition, many countries reduced rehabilitation treatments for outpatients with chronic diseases and disabilities in response to social distancing policies implemented to reduce the spread of infection through the population (21). Among the mitigation measures that health systems faced was the rapid implementation of remote rehabilitation programs in the population with COVID-19 and other non-COVID pathologies (21, 22). The literature has shown their effectiveness to be similar to face-to-face programs (22).

The rehabilitation might have a key role in improving functional outcomes of COVID-19 sequelae and can reduce the length of hospital stay and improve health outcomes (23, 24). The literature reports the effect of multimodal cardiopulmonary rehabilitation with an improvement in outcomes measures, independent of previous ventilation status (25). Therefore, the evidence pointed out that COVID-19 patients might have sequelae that need rehabilitative interventions in outpatient clinics or remote rehabilitation (23). The possible mechanisms that support the rehabilitation in post-COVID-19 patients are not yet fully elucidated (26). One possible explanation is that exercise improves the neuromotor function, which would explain the improvement in physical tests in this population (26). Another possible explanation is that the functionality of most individuals was still severely restricted at the time-point of admission, especially in subjects under mechanical ventilation (27, 28). These factors, added to the fact that the most severe patients have especially cardiometabolic comorbidities, cause the disease to have serious sequelae, and therefore, the potential for response to rehabilitation is greater (26).

Undoubtedly, the heterogeneity of government responses and the different preparation of health systems has determined that the pandemic's impact differs from country to country. The burden of COVID-19 is far higher in developing countries, reflecting a combination of elevated transmission to middle-aged and older adults and limited access to adequate healthcare (29).

Chile is a developing country with a long north-south extension, and is socially, culturally, and ethnically diverse. Despite being considered a developing country, the Chilean population has a very accessible, integrated public health system characterized as high performance by the World Health Organization (WHO) (30). The Chilean health system consists of public and private sectors coordinated by the Ministry of Health (MINSAL) and its different departments (31).

The different MINSAL departments intervene in public policies to face COVID-19, with a specific rehabilitation and disability department directing, coordinating and integrating the public rehabilitation policies from the primary care centers to the intensive care unit (ICU). Chile is no stranger to the challenges posed by rehabilitation in a developing country. Therefore, our principal objective was to describe the approach to rehabilitation during the pandemic in Chile in the public health system since rehabilitation is considered a relevant health strategy from the prevention to management of complications, mitigation of sequelae, or new complications associated with COVID-19.

## Methods

A descriptive study was conducted on the rehabilitation strategies implemented by MINSAL to respond to the COVID-19 pandemic based on the documents and protocols developed by the Department of Rehabilitation and Disability available in its virtual library. For this, the MINSAL website was reviewed, specifically the virtual library of the department of rehabilitation and disability (https://rehabilitacion.minsal.cl/biblioteca/biblioteca-rehabilitacion/) up to February 2022. We report intervention characteristics as suggested by the Template for Intervention Description and Replication for Population Health and Policy (TIDieR-PHP) (32).

**Figure 1:**
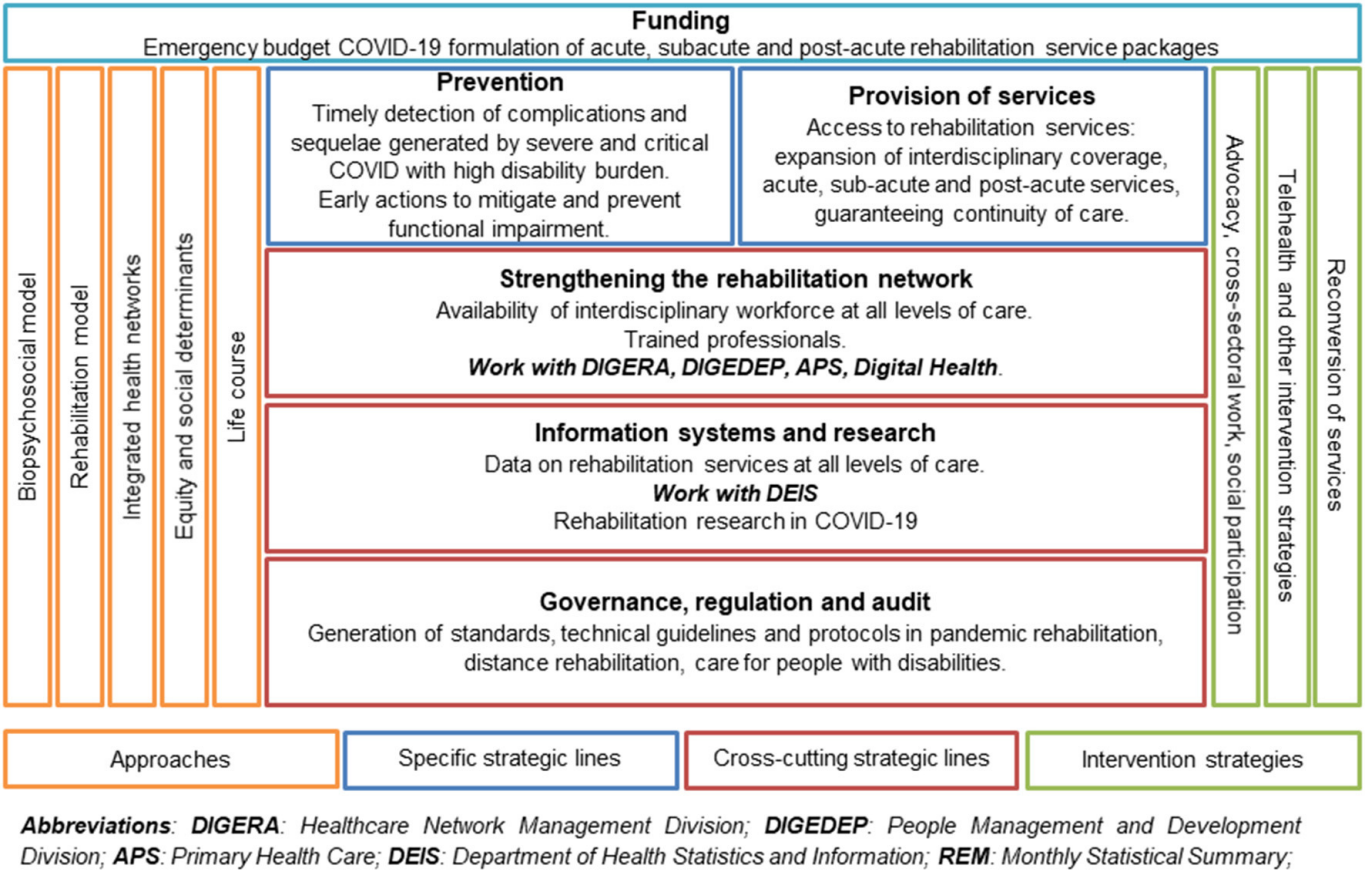
Matrix of access to rehabilitation services during the COVID-19 pandemic.

The analysis begins with the context of the Chilean health system and the structure in which rehabilitation services are located. Then, and considering that at the moment the pandemic was declared, MINSAL's Department of Rehabilitation and Disability had generated a matrix of access to rehabilitation services in COVID-19, the analysis of all the information collected was organized according to the five central axes considered: (1) approaches, referred to the rehabilitation care model implemented in Chile and how it integrates with integrated health networks throughout the life course; (2) specific strategic lines, referring to how the health system acted preventively to detect sequelae and complications and how actions were taken to mitigate these sequelae and guarantee continuity of care; (3) transversal strategy lines, referring to how the rehabilitation network was strengthened through interdisciplinary work, together with the generation of information and evidence by the different health institutions involved; (4) intervention strategies, referring to how the information was generated and disseminated through the health network and (5) funding, referring to how the resources that financed the interventions were obtained (Figure 1). This algorithm was developed to be applied at all health levels, from primary care to the ICU.

Additionally, three authors reviewed and analyzed the data (RT-C, RG-A, PS) of: (1) admissions to rehabilitation programs between January 2018 and October 2021 (pre and post-pandemic) in primary care, outpatient care, low complexity hospitalized and hospitalized in an ICU, and (2) confirmed cases, deaths, and cases diagnosed with a post-COVID-19 condition between March 2020 and December 2021. This information was obtained from the Department of Health Statistics and Information (DEIS) and EPI-VIGILA, a private informational project to facilitate access to information from health centers that require interaction with the Epidemiology Department of the MINSAL (https://epivigila.minsal.cl/).

## Results

### Context

In 2005, within the framework of the Health Reform, MINSAL published the “Comprehensive Health Care Model”. This reports on the reflections and agreements of authorities, academics, and health teams about the new health care model, which promotes and facilitates efficient, effective, and timely care. Such care is directed to more than the patient or the disease as isolated events, but rather it considers people physically and mentally as a whole, as social beings belonging to different families and communities, who are in a permanent process of integration and adaptation to their physical, social and cultural environment (33). Three essential principles are declared in the health system based on primary care: people-centered, comprehensiveness, and continuity of care.

In this context, rehabilitation actions are undertaken in:

- Primary care: There are currently 285 community-based rehabilitation centers. In 2018, according to the national database, 278,739 cases were admitted to rehabilitation, with the main diagnoses being: painful syndromes of traumatic origin, non-traumatic origin, mild-moderate knee, hip osteoarthritis, and stroke (34).
- Hospitals: The rehabilitation services are coordinated by each hospital's physical medicine and rehabilitation units. According to the national database, in 2018, 246,253 people were admitted to hospital rehabilitation, 117,388 to rehabilitation in open care, 20,541 to rehabilitation in ICU, and 101,490 to rehabilitation in medium and basic care. The main diagnoses were musculoskeletal lesions, chronic respiratory disease, and stroke (34).

In the Chilean health system, rehabilitation is incorporated throughout the entire disease process, including rehabilitation teams in the ICU that work from early mobilization through to transfer to other units or discharge, rehabilitation in hospital with inpatients at several clinical services as well as with outpatients, and in the primary care where the actions increase their coverage and are installed in community rehabilitation or family health centers under the community-based rehabilitation model. Rehabilitation is based on an individualized treatment plan, structured in multidisciplinary programs focused on functional objectives and duration according to the recommendations of specific clinical guidelines or current knowledge.

### Epidemiology of COVID-19 in Chile

Until March 15, 2022, a total of 3,313,242 cases of COVID-19 had been confirmed in Chile, of which 44,005 cases died (1.33%), ranking sixth in Latin America after Brazil, Argentina, Colombia, Mexico and Peru (35, 36). Regarding vaccination, 89.96% of the population has vaccination with two doses, making Chile second in the world with more than 10 million inhabitants with the largest immunized population, the largest immunized population in Latin America and with 77.4% having had a booster dose (35). As for people projected with a diagnosis of post-COVID-19 condition, until December of 2021, there are 80,528, i.e., 4.86% of the total confirmed cases (Table 1).

**Table 1:** Cases of post-COVID-19 conditions during 2020–2021.

**Month, year**	**Confirmed cases**	**Deaths**	**Post-COVID-19 condition**
March, 2020	2,737	17	120
April, 2020	13,285	238	426
May, 2020	83,609	2,042	2,138
June, 2020	140,872	4,775	5,501
July, 2020	76,144	2,759	2,791
August, 2020	56,049	1,732	2,360
September, 2020	51,171	1,480	2,259
October, 2020	47,143	1,392	2,052
November, 2020	41,487	1,177	1,854
December, 2020	57,211	1,230	2,961
January, 2021	118,095	1,998	6,664
February, 2021	98469	2102	5110
March, 2021	170,583	2,791	9,723
April, 2021	201,480	3,109	11,676
May, 2021	186,264	2,986	10,680
June, 2021	172,249	3,429	9,192
July, 2021	60,022	2,558	1,808
August, 2021	22,915	1,268	392
September, 2021	26,280	609	1,315
October, 2021	15,667	364	782
November, 2021	9,301	216	465
December, 2021	5,182	120	259
Total	1,656,215	38,392	80,528

### Approaches

The rehabilitation model is based on the biopsychosocial model which dynamic interpersonal, biological, and psychological systems interacting with contextual factors to shape health over the life span (37). Second, it is based on the integrated networks proposed by the WHO/PAHO, and which have been described as one of the critical health challenges in Latin America (38). Third, it is based on social determinants, defined as the social conditions in which people live and work that directly or indirectly influence the health and disease process (39). The biopsychosocial model provides a useful perspective for understanding the development and characteristics of the COVID-19 pandemic and its anticipated long-term consequences for society as well as individuals (40). Finally, although the consequences of COVID-19 have been seen primarily in adults, the approach is intended for the entire life cycle.

### Cross-Cutting Strategic Lines

The lines of transversal development consist of three axes. The first is strengthening the rehabilitation network, which is based on the availability of an interdisciplinary workforce at all health levels. These professionals are trained in rehabilitation and adapt to the functional needs of post-COVID-19 patients. At this level, there is a close relationship with the Healthcare Network Management Division (DIGERA), the People Management and Development Division (DIGEDEP), Primary Health Care (APS), and Hospital Digital (Supplementary Material 1).

The second axis is based on the use of information and evidence systems from all levels of care. For this, the MINSAL has the DEIS, which includes information on the coverage of different health conditions. Particularly for COVID-19, a specific monthly statistical summary was created (REM A-28) including remote rehabilitation, which was defined as the delivery of therapeutic rehabilitation at a distance or offsite using telecommunication technologies (41).

Finally, the third axis, based on the stewardship, regulation, and supervision, is responsible for the generation of standards, technical guidelines, and rehabilitation protocols in the pandemic. National innovators in rehabilitation were invited together with representatives of scientific societies and professional associations to prepare these documents. They also coordinate remote rehabilitation and care for people with disabilities and a post-COVID-19 condition. This axis works with the subsecretary of social security (SUSESO).

In particular, the Department of Rehabilitation and Disability has created a series of support documents for professionals in the health network (Table 2). All the documents followed the recommendations issued by the WHO regarding the maintenance of essential services, including rehabilitation (42). Additionally, Chile participated in the WHO consultations regarding the health care continuity surveys, in which one of the aspects evaluated was rehabilitation (43).

**Table 2:** Documents related to rehabilitation generated by MINSAL.

**Document**	**Access link**	**Release date**
Rehabilitation 2030 a call to action	https://rehabilitacion.minsal.cl/rehabilitacion-2030-un-llamado-a-la-accion/	October 2018
Rehabilitation in health systems	https://rehabilitacion.minsal.cl/rehabilitacion-en-los-sistemas-de-salud/	October 2018
Rehabilitation protocol for severe and critical COVID-19 from acute to post-acute stage	https://diprece.minsal.cl/wp-content/uploads/2020/09/Protocolo-de-Rehabilitacio%CC%81n-en-personas-COVID-19-grave-y-cri%CC%81tico.-Desde-la-etapa-aguda-a-la-post-aguda.pdf	September 2020
Technical guidelines for rehabilitation in times of pandemic COVID-19 prevention post COVID syndrome	https://diprece.minsal.cl/wp-content/uploads/2020/09/Orientaciones-Te%CC%81cnicas-para-la-Rehabilitacio%CC%81n-en-tiempos-de-pandemia.-Prevencio%CC%81n-del-Si%CC%81ndrome-post-COVID.pdf	September 2020
Health residency strategy recommendations for the admission, follow-up and discharge of people with disabilities	https://diprece.minsal.cl/wp-content/uploads/2020/10/ESTRATEGIA-DE-RESIDENCIAS-SANITARIAS_RECOMENDACIONES-PARA-EL-INGRESO-SEGUIMIENTO-Y-EGRESO-DE-PERSONAS-CON-DISCAPACIDAD.pdf	October 2020
Recommendations for daily cleaning and disinfection of technical aids in the COVID-19 context.	https://rehabilitacion.minsal.cl/wp-content/uploads/2020/05/Recomendaciones-para-la-limpieza-y-desinfecci%c3%b3n-diaria-de-ayudas-t%c3%a9cnicas-en-el-contexto-del-COVID-19_v3.pdf	May 2020
Recommendations for the suspicion, diagnosis and rehabilitation of people affected by persistent and prolonged COVID-19 (Long-COVID)	https://rehabilitacion.minsal.cl/wp-content/uploads/2020/09/Recomendaciones-long-COVID-final.pdf	September 2020
Technical guidelines for rehabilitation in times of pandemic. Prevention of post-COVID syndrome.	https://rehabilitacion.minsal.cl/wp-content/uploads/2020/09/Orientaciones-Te%cc%81cnicas-para-la-Rehabilitacio%cc%81n-en-tiempos-de-pandemia.-Prevencio%cc%81n-del-Si%cc%81ndrome-post-COVID.pdf	September 2020
Rehabilitation protocol for severe and critical COVID-19 patients. From acute to post-acute stage.	https://rehabilitacion.minsal.cl/wp-content/uploads/2020/09/Protocolo-de-Rehabilitacio%cc%81n-en-personas-COVID-19-grave-y-cri%cc%81tico.-Desde-la-etapa-aguda-a-la-post-aguda.pdf	September 2020
National rehabilitation model	https://rehabilitacion.minsal.cl/wp-content/uploads/2022/03/Plan-Nacional-de-RH-en-COVID-19-Chile.pdf	March 2022

### Specific Strategic Lines

The specific lines rest on two pillars. The first, prevention, refers to the timely detection of complications and sequelae generated by severe and critical COVID-19 with a high burden of disability. It is oriented to delivering early actions to mitigate and prevent alterations in functionality. The second pillar is the provision of rehabilitation services by expanding interdisciplinary coverage, with benefits in the acute, sub-acute, and post-acute stages, guaranteeing the continuity of care from the hospital (including the ICU) to outpatient rehabilitation with primary care.

### Intervention Strategies

The intervention strategy is based on generating and disseminating information throughout the entire health network. The intervention recommendations developed by MINSAL have been made from the available evidence, through the generation of own documents and with strict integration with the documents generated by the WHO (42). In addition, minimum standards of quality of services have been established. This is backed by advocacy and close cross-sector work.

### Funding

The resources were obtained through the COVID-19 emergency budget that allowed the hiring of rehabilitation human resources based on rehabilitation service packages worked in closed care and outpatient care.

Supporting the five axes mentioned above, a digital strategy called Hospital Digital (www.hospitaldigital.cl) was implemented. This is a web platform and part of the digital channels available to clinicians and citizens to deliver information and support remotely, with national coverage, 24 h a day, 7 days a week. Hospital Digital is a new model of patient-focused health care, and it takes advantage of the potential of technologies to bring care to people, providing an alternative to the traditional model (based on a network of physical establishments and with schedule restrictions). Thus, it is expected to transform and modernize the public health system. Additionally, this platform allows the transfer of knowledge of prevention and promotion to the entire health network.

### Rehabilitation Response

The annual increase in the average number of ICU beds in the pre-pandemic period in the country was 3% per year. The increase in hospital capacity paralleled the increase in health professionals, including professionals working in rehabilitation, in this case, in the ICU.

**Figure 2:**
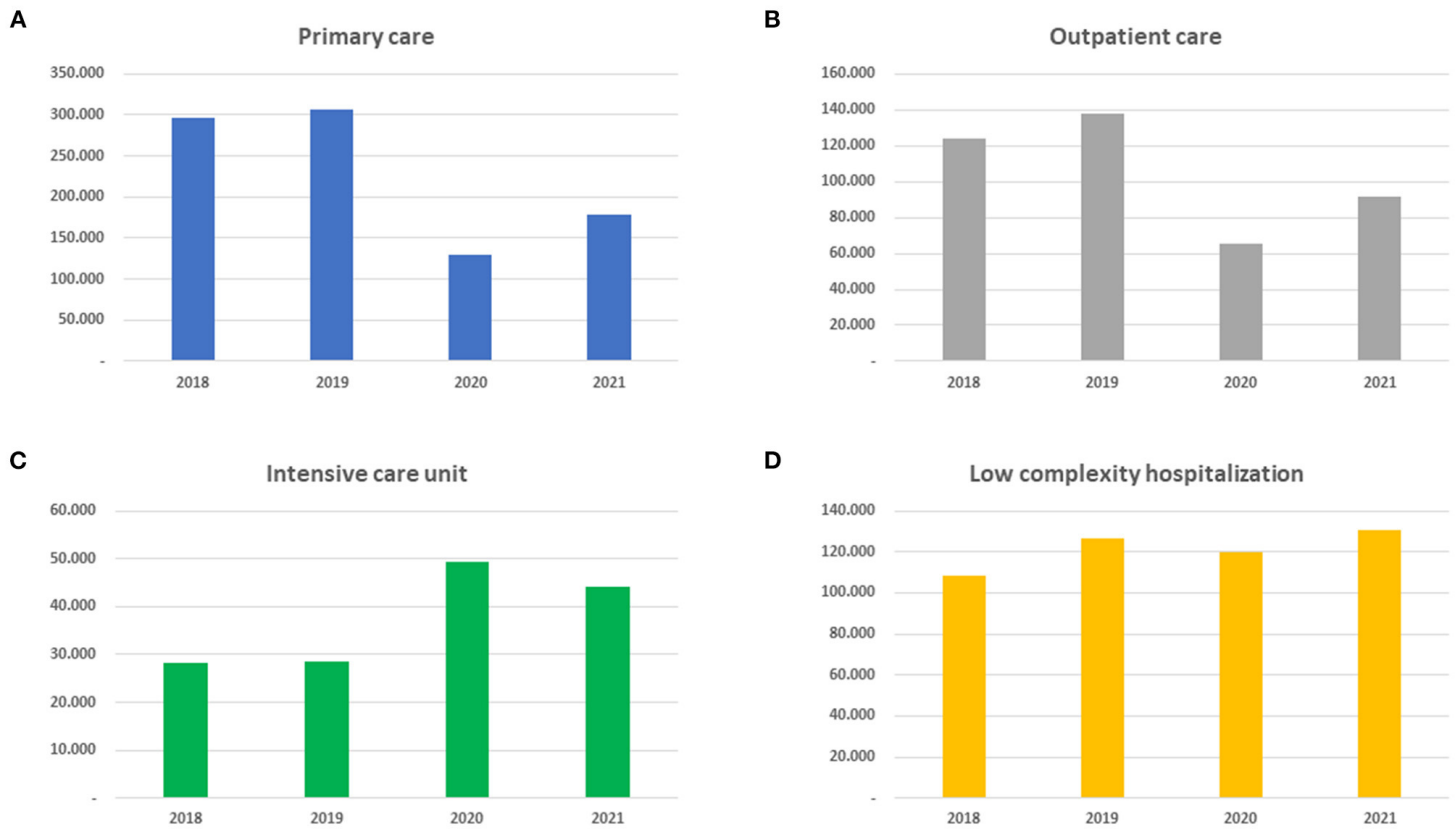
**(A–D)** Admission of patients according to level of complexity.

We reviewed the information provided by the DEIS that showed a decrease from 306,242 to 129,494 admissions to primary care. In outpatient care, the decrease was from 138,043 to 65,583, and admissions to medium and low complexity hospitalization decreased from 126,245 to 119,805. However, the admission of people in ICU rehabilitation increased from 28,600 to 49,248 patients, representing an increase of 68% (Figure 2).

For outpatient care in secondary and tertiary care centers, 151,436 remote rehabilitation interventions were performed during the year 2020 (July-December) and 194,447 between January and August 2021 (34).

The response of primary care was variable depending on the epidemiological context. Professionals were relocated to follow up active cases and in the mass vaccination process.

## Discussion

We showed that it is possible to implement a national rehabilitation policy that integrates all levels of health care in response to a pandemic in a developing country. Although it is still too early to evaluate the effectiveness of the rehabilitation plan in COVID-19 in Chile, from a scientific point of view, experiences in evaluation and intervention in these patients have been reported, both at the hospital level and in primary care (26, 44–46).

An important aspect that influenced the rapid implementation of the national rehabilitation policies was having the first national rehabilitation plan that could be put into practice (34). Although the gradual implementation of this plan would have been better for the different conditions that affect people's functioning, the emergency of this pandemic was a litmus test to assess the system's response and the applicability of this national rehabilitation plan to a stressful situation.

The viability of a national plan of this magnitude in a developing country needs a certain amount of funding for its implementation which is not always available. In this case, the consideration of rehabilitation as an essential service by the WHO was vital. At the same time, the incorporation of non-governmental institutions such as academic or scientific institutions is also needed. In this sense, scientific societies generated documents and educational strategies that contribute at the same time as providing better quality standards to the population that requires rehabilitation (47).

From the point of view of the number of admissions, this decreased in primary care. This reduction was in line with the impact on rehabilitation services reported by the WHO and has several possible explanations (42). One is the mobility restriction measures implemented by many countries (48). Another is that health professionals had to collaborate in traceability and vaccination teams. Yet admissions were not totally eliminated since many teams continued to provide remote care with support from MINSAL in pathologies where the evidence showed favorable results with remote rehabilitation (22).

Our data estimated that ~7.5% of patients will develop a post-COVID-19 condition, but this data should be analyzed with caution, since the definition of a post-COVID-19 condition is recent and at that point there was no consensus among the different terms used previously and how to estimate the prevalence (11, 13).

One of the strengths of implementing public policies in rehabilitation was that the professionals were prepared from a technical point of view. This was given by rehabilitation network throughout the country. Obviously, without such human resources, it would have been more challenging to implement a policy from scratch.

Another strength that we must highlight is the rapid adaptation of the system to deliver remote attention. Important support for this decision is that remote rehabilitation has shown similar effects as face-to-face care (22). This was strongly supported by the Hospital Digital platform that contributed to training in this disease and was a permanent support point for the health network in case of doubts.

Another critical point to consider is the influence of social determinants. Our country is not alien to this reality, and it has already been shown that these social determinants contributed to greater alteration in functioning and higher mortality in the hospitalized population (49, 50). One of the ways to reduce this effect is the implementation of these policies at the national level involving public and private health, favoring access to the most vulnerable population.

### Limitations

An important limitation of this study is that it is a descriptive analysis from the perspective of the national health authority. Undoubtedly in the future, evaluation by independent organizations will be needed to assess how this plan was received by the different actors in the health system. On the other hand, this approach incorporates only the public health system, not the private one. However, it is important to emphasize that the ICU beds of the private system were incorporated into the integrated public care network.

## Conclusion

A developing country has managed to coordinate a rehabilitation policy for people with COVID-19 by generating a structure comprised of the different levels, including critical, post-acute, or post-COVID-19 clinic scale-up of rehabilitation. However, the effectiveness of this policy will need to be evaluated in the future. Similar countries, particularly in Latin America, can use this information to establish policies adapted to their local reality and thus face the burden of post-COVID-19 sequelae through rehabilitation.

## Data Availability Statement

Publicly available datasets were analyzed in this study. This data can be found at: https://rehabilitacion.minsal.cl.

## Author Contributions

RT-C, RG-A, and PS designed the study methodology, conducted formal analysis, investigation, data curation, and reviewed and edited the manuscript. RT-C wrote the original draft. XN-Z and KH-M conceptualized the study and validated the data. RV-S, CC-A, MR-Q, and CÁ-V provided and validated the data. All authors reviewed critically the manuscript and approved the final version.

## Funding

This work was supported by a service provision from the Universidad de La Frontera to the Chilean Ministry of Health. OC 757-2019-SE21.

## Conflict of Interest

XN-Z, KH-M, RV-S, CC-A, MR-Q, and CÁ-V are workers of the Ministry of Health of Chile, however, they did not work directly in the analysis and interpretation of the data. The remaining authors declare that the research was conducted in the absence of any commercial or financial relationships that could be construed as a potential conflict of interest.

## Publisher's Note

All claims expressed in this article are solely those of the authors and do not necessarily represent those of their affiliated organizations, or those of the publisher, the editors and the reviewers. Any product that may be evaluated in this article, or claim that may be made by its manufacturer, is not guaranteed or endorsed by the publisher.
