# Supplementary material for: How a Developing Country Faces COVID-19 Rehabilitation: The Chilean Experience
Source: Front Public Health. 2022 Jul 6;10:924068. doi: 10.3389/fpubh.2022.924068 (PMC9298948; doi:10.3389/fpubh.2022.924068)
Supplement: Supplementary file 1 [file Table_1.DOCX]

Supplementary Material

## Supplementary Figure


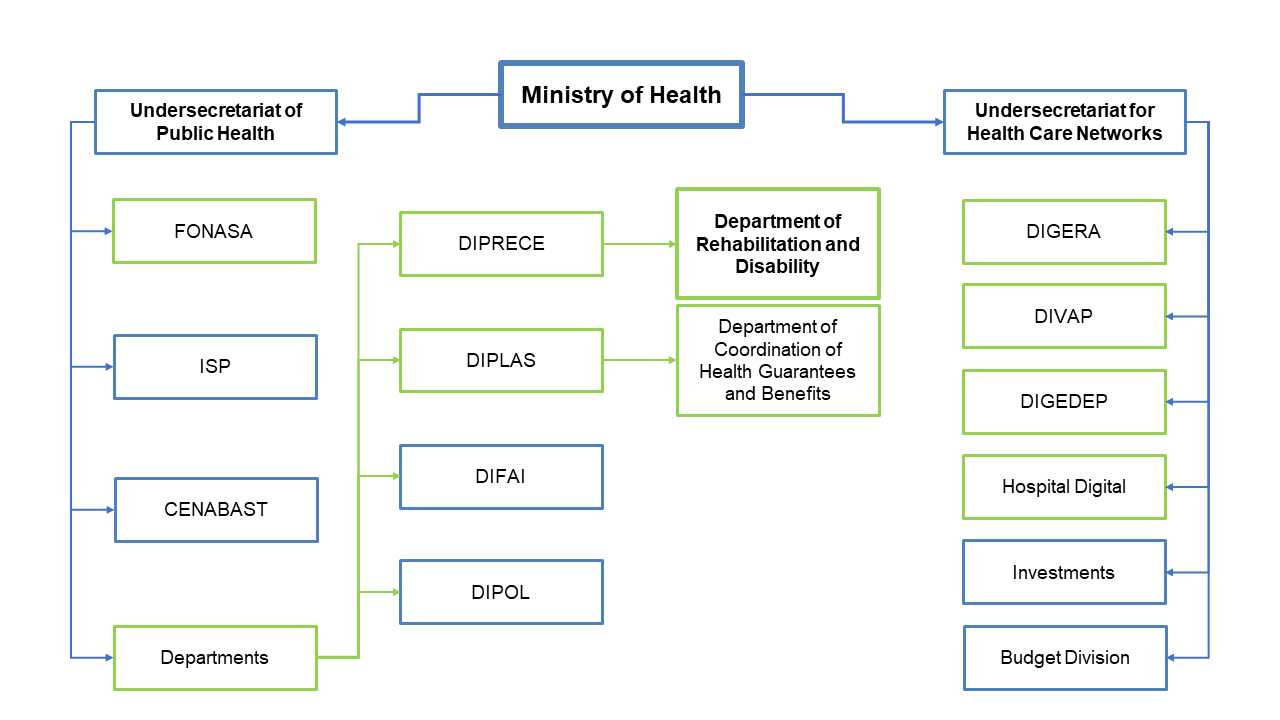


***Abbreviations****:*

***FONASA****: Fondo Nacional de Salud (National Health Fund)*

***ISP:*** *Instituto de Salud Pública (Public Health Institute)*

***CENABAST:*** *Central de Abastecimiento del Sistema Nacional de Servicios de Salud (Central Supply Center of the National Health Services System)*

***DIPRECE:*** *División de Prevención y Control de Enfermedades (Disease Prevention and Control Division)*

***DIPLAS:*** *División de Planificación Sanitaria (Health Planning Division)*

***DIFAI:*** *División de Finanzas y Administración Interna (Finance and Internal Administration Division)*

***DIPOL:*** *División de Políticas Públicas (Public Policy Division)*

***DIGERA****: División de Gestión de Redes Asistenciales (Healthcare Network Management Division)*

***DIVAP:*** *División de Atención Primaria (Primary Care Division)*

***DIGEDEP****: División de Gestión y Desarrollo de las Personas (People Management and Development Division)*

**Supplementary Figure 1.** Abbreviated organization chart of the Chilean Ministry of Health, showing the main units articulated with the Department of Rehabilitation and Disability.
